# Bacteriological and Immunological Profiling of Meconium and Fecal Samples from Preterm Infants: A Two-Year Follow-Up Study

**DOI:** 10.3390/nu9121293

**Published:** 2017-11-27

**Authors:** Marta Gómez, Laura Moles, Irene Espinosa-Martos, Gerardo Bustos, Willem M. de Vos, Leónides Fernández, Juan M. Rodríguez, Susana Fuentes, Esther Jiménez

**Affiliations:** 1 Departamento de Nutrición, Bromatología y Tecnología de los Alimentos, Universidad Complutense de Madrid, 28040 Madrid, Spain; marta_gmz@hotmail.com (M.G.); lawita21@hotmail.com (L.M.); leonides@ucm.es (L.F.); jmrodrig@ucm.es (J.M.R.); 2 Servicio de Pediatría Hospital Francesc de Borja, 46702 Gandía, Valencia, Spain; 3 Probisearch, SLU, Tres Cantos, 28760 Madrid, Spain; irenee70@gmail.com (I.E.-M.); 4 Servicio de Neonatología, Hospital Universitario 12 de Octubre, 28041 Madrid, Spain, Red SAMID; gerardo.bustos@salud.madrid.org; 5 Laboratory of Microbiology, Wageningen University, 6708 PB Wageningen, The Netherlands; willem.devos@wur.nl; 6 Department of Bacteriology and Immunology, University of Helsinki, 00100 Helsinki, Finland

**Keywords:** prematurity, infant gut microbiota, DNA microarray, immune maturation

## Abstract

An abnormal colonization pattern of the preterm gut may affect immune maturation and exert a long-term influence on the intestinal bacterial composition and host health. However, follow-up studies assessing the evolution of the fecal microbiota of infants that were born preterm are very scarce. In this work, the bacterial compositions of fecal samples, obtained from sixteen 2-year-old infants were evaluated using a phylogenetic microarray; subsequently, the results were compared with those obtained in a previous study from samples of meconium and feces collected from the same infants while they stayed in the neonatal intensive care unit (NICU). In parallel, the concentration of a wide range of cytokines, chemokines, growth factors and immunoglobulins were determined in meconium and fecal samples. Globally, a higher bacterial diversity and a lower interindividual variability were observed in 2-year-olds’ feces, when compared to the samples obtained during their first days of life. Hospital-associated fecal bacteria, that were dominant during the NICU stay, seemed to be replaced, two years later, by genera, which are usually predominant in the healthy adult microbiome. The immune profile of the meconium and fecal samples differed, depending on the sampling time, showing different immune maturation statuses of the gut.

## 1. Introduction

The microbial composition of the gastrointestinal tract in humans undergoes remarkable changes in our life span [1,2,3]. The most dynamic period of changes in human intestinal microbiota is reported after birth, when the scarce bacteria present in the intrauterine environment make space for a complex microbial community. The conventional belief is that the development of the intestinal microbiota in term infants involves early colonization by facultative anaerobes, which generate a reducing environment, favoring the growth of strictly anaerobic bacteria [4,5,6,7]. From an initial low diversity and low complexity, the intestinal bacterial community of the infant will gradually develop and mature, reaching an enduring adult state after 2–3 years of age [6,8,9].

However, the establishment and development of the intestinal microbiota differ between preterm and healthy term infants [10,11]. In the former, several factors, including mother/infant antibiotic therapy, Caesarean section, early separation from parents, delayed enteral feeding, invasive medical procedures or a long stay in the neonatal intensive care unit (NICU), are assumed to exert strong influences on early colonization of the infant gut [5,12,13]. Previous studies monitoring the bacterial communities, using culture-dependent and independent techniques, in term and preterm infants, have detected a reduced number of bacterial species in the fecal microbiota of preterm infants, compared with term infants [14,15,16]. In healthy, full-term, vaginally-delivered newborns, gut microbial colonization is initiated with facultative anaerobic microorganisms, which decrease the intestinal redox potential, helping the subsequent establishment of strict anaerobic microorganisms, such as *Bifidobacterium*, *Bacteroides* or *Clostridium* [8,17]. In preterm infants, the establishment of obligate anaerobes, especially bifidobacteria, are delayed, compared with full-term infants and facultative anaerobes, such as enterobacteria, enterococci and staphylococci, seem to persist for several weeks at high levels in the preterm infant’s fecal microbiota [14,15,18,19,20]. The abnormal gut colonization in preterm infants during their first weeks of life [14,17,21] may affect the maturation of the gut barrier as well as its nutritional and immunological functions at that time and later [22,23].

There is circumstantial evidence that initial microbial gut colonization and the resulting immune and metabolic programming could have a long-lasting influence on the risk for future diseases [6,24]. However, little is known about the possible influence of gut microbiota on the human immune system and how early bacterial colonization affects immune maturation [25,26], particularly among preterm infants [16]. Several studies have assessed immune compounds in saliva, umbilical cord blood or peripheral blood of infants [25,27,28,29,30,31], but few have described the presence of cytokines, chemokines, growth factors or immunoglobulins in fecal samples of preterm babies [32,33,34,35].

In this context, the objectives of this study were, firstly, to study if the abnormal initial colonization of preterm babies previously studied [36] may affect their fecal bacterial composition when they are 2 years old, by using a phylogenetic microarray [37] and, secondly, to characterize and compare the immune profiles of the meconium and infant feces, obtained from such infants in the first weeks after birth and, also, at the age of 2.

## 2. Materials and Methods

### 2.1. Patients and Sampling

This prospective study included sixteen 2-year-old infants, who were born prematurely at the Hospital Universitario 12 de Octubre, Madrid (Spain) (Table 1).

Written informed parental consent was obtained for each infant before inclusion in the study, which was approved by the Ethical Committee on Clinical Research of the Hospital Clínico San Carlos of Madrid (10/017-E). This study was conducted in accordance with the Declaration of Helsinki. Characteristics to be eligible for enrolment have been described previously [32]. Relevant clinical data recorded for each infant, such as length of antibiotic therapy, parenteral nutrition, nasogastric feeding, mechanical ventilation, hospital stay and type of feeding, are described in Table 2. All infants were fed with human milk (donor milk and/or their own mother’s milk) and, occasionally, with preterm formula.

The medical staff from the Department of Neonatology of the hospital had collected first spontaneously evacuated meconium and fecal samples during the infants’ stays at the NICU [36]. Later, when the infants reached 2 years of age, parents were contacted to provide an additional fecal sample, if their infants had not taken antibiotics within the previous 2 months. All the samples were stored at −20 °C until analysis and processed as described previously [36].

### 2.2. Human Intestinal Tract Chip (HITChip) Analysis

DNA extraction from fecal samples was performed following the protocol described in Moles et al. (2013) [36]. All the steps for the HITChip microarray analysis, including polymerase chain reaction (PCR) amplification of 16S rRNA genes, RNA production and labeling, hybridization and data extraction, were performed as described previously [33]. Then, the PCR products were purified, using the High Pure PCR Product Purification kit (Roche, Mannheim, Germany), according to the manufacturer’s instructions. In vitro transcription of the T7 promoter-carrying 16S rRNA genes was performed using the Riboprobe System (Promega, Madison, WI, USA) while amino-allyl-modified nucleotides were coupled with CyDye using the Post-Labeling Reactive Dye (Amersham Biosciences, Little Chalfont, UK).

Data were extracted from the microarray images, using Agilent Feature Extraction software, version 9.1 (http://www.agilent.com), subsequently normalized, and further analyzed using a set of R-based scripts (http://r-project.org) in combination with a custom-designed relational database that runs under the MySQL database management system. Hierarchical clustering of probe profiles was carried out using the Pearson’s distance and Ward’s minimum variance method. Normalized hybridization signals for 23 Level 1 (phylum-like) groups and 131 Level 2 (genus-like) groups, as defined previously [37], are available as Supplementary Materials in Tables S1 and S2, respectively.

Data, available on the HITChip database, on fecal samples from 2–4-year-old healthy children and healthy adults were used for comparison purposes. Demographic data and other information from the 2–4-year-old healthy children has been published previously [38]. The bacterial compositions of fecal samples from healthy adult subjects were selected from the CO-MIC cohort, a study of the intestinal microbiome among Irritable Bowel Syndrome patients and healthy individuals. None of the individuals selected received antibiotics.

### 2.3. Immunological Analysis

The concentrations of 18 cytokines, chemokines, and growth factors, including interleukins (IL) 1_β_, 2, 4, 5, 6, 7, 8, 10, 12(p70), 13, and 17, interferon-gamma (IFN-γ), granulocyte colony stimulating factor (G-CSF), granulocyte-macrophage colony stimulating factor (GM-CSF), growth regulated oncogene-alpha (Gro-α), monocyte chemoattractant protein-1 (MCP-1), macrophage inflammatory protein-1_β_ (MIP-1_β_) and tumor necrosis factor-alpha (TNF-α), were determined in meconium and fecal samples by using the Human Cytokine group I and II assay kits (Bio-Rad Laboratories Inc., Hercules, CA, USA) in a Bioplex 200 system instrument (Bio-Rad). Briefly, 0.1 g of fecal samples was suspended in 1 mL of PBS. After homogenization, the samples were centrifuged (14,000× *g*, 15 min, 4 °C) and the supernatants (≥200 μL) were collected. Determinations in meconium and fecal samples were carried out in duplicate.

The concentrations of immunoglobulin (Ig) G1, IgG2, IgG3, IgG4, IgM and IgA in the samples were determined using the Bio-Plex Pro Human Isotyping Assay kit (Bio-Rad) using the Bioplex 200 system. For this purpose, the samples were conditioned, as described above, for cytokine analysis. All analyses were carried out in duplicate, following the manufacturer’s protocol.

Calibration curves for each analyte were constructed using triplicate values for each known concentration and the Bio-Plex Manager 6.0 software (Bio-Rad, Hercules, CA, USA).

### 2.4. Statistical Analysis

Quantitative data were expressed as the mean and 95% confidence interval (CI) of the mean or, when they were not normally distributed, as the median and interquartile range (IQR). The richness and diversity of the meconium and fecal microbiota were determined by calculating the Shannon–Weaver diversity index, which takes into account the number and evenness of the bacterial species. Friedman’s non-parametric repeated measures comparisons were applied to determine differences between the hybridization signal intensities of genus-like bacterial groups across time. Chi-square independency tests were used to evaluate differences in the detection frequencies of immune compounds. One-way ANOVA or Kruskal–Wallis tests were used to compare differences in the concentrations of immune compounds at different sampling times. The immune compounds were further analyzed, applying exploratory multivariate analyses, such as the principal component analysis (PCA), multiple discriminant analysis (MDA) and cluster analysis (CA). Differences were considered significant at *p* < 0.05. Statgraphics Centurion XVI version 16.1.15 (Statpoint Technologies Inc., Warrenton, VA, USA) and R 2.13.2 (R Foundation for Statistical Computing, https://www.r-project.org, Vienna, Austria) software were used to carry out the analyses cited above.

For comprehensive multivariate statistical analyses, Canoco software for Windows 5.0 (Wageningen, The Netherlands) was used [34]. A redundancy analysis was performed to assess correlations between the microbial groups detected by the HITChip and the sample characteristics. The log-transformed hybridization signals of 130 genus-level phylogenetic groups targeted by the HITChip were used as biological variables. Gestational age, gender, birth weight, Z score, vaginal vs. cesarean section, age, antibiotics (mother and/or infant), time of first passage of meconium, type of nutrition, time of enteral and parenteral nutrition, sepsis and hospital stay were included as explanatory variables. The Monte Carlo Permutation testing (MCPT) was used to assess the significance of the variation in large data sets.

To evaluate the significance of the difference between datasets not-normally distributed, *p* values were calculated by Wilcoxon rank sum tests.

## 3. Results

### 3.1. Characteristics of the Infants

The 16 infants that participated in this study had, at birth, a mean gestational age of 28 weeks (ranging from 24 to 32 weeks) and a mean birth weight of 1220 g (ranging from 600 to 2030 g) (Table 1). Half of the infants (*n* = 8) were born by Cesarean section. All of them, except one, received antibacterial prophylaxis at least for the first 3 days of life, and nine needed mechanical ventilation (Table 2). Infants were fed either with their own mother’s milk, donor milk and/or preterm formula by nasogastric feeding tube for, at least 21 days after delivery. The time required for spontaneous delivery of the first meconium oscillated between the first minutes to day 6 after birth. In addition to the meconium samples, fecal samples were available from the same infants, obtained after 3 weeks (±21 days) and 2 years (±730 days) after birth.

### 3.2. HITChip Analysis

**Figure 1:**
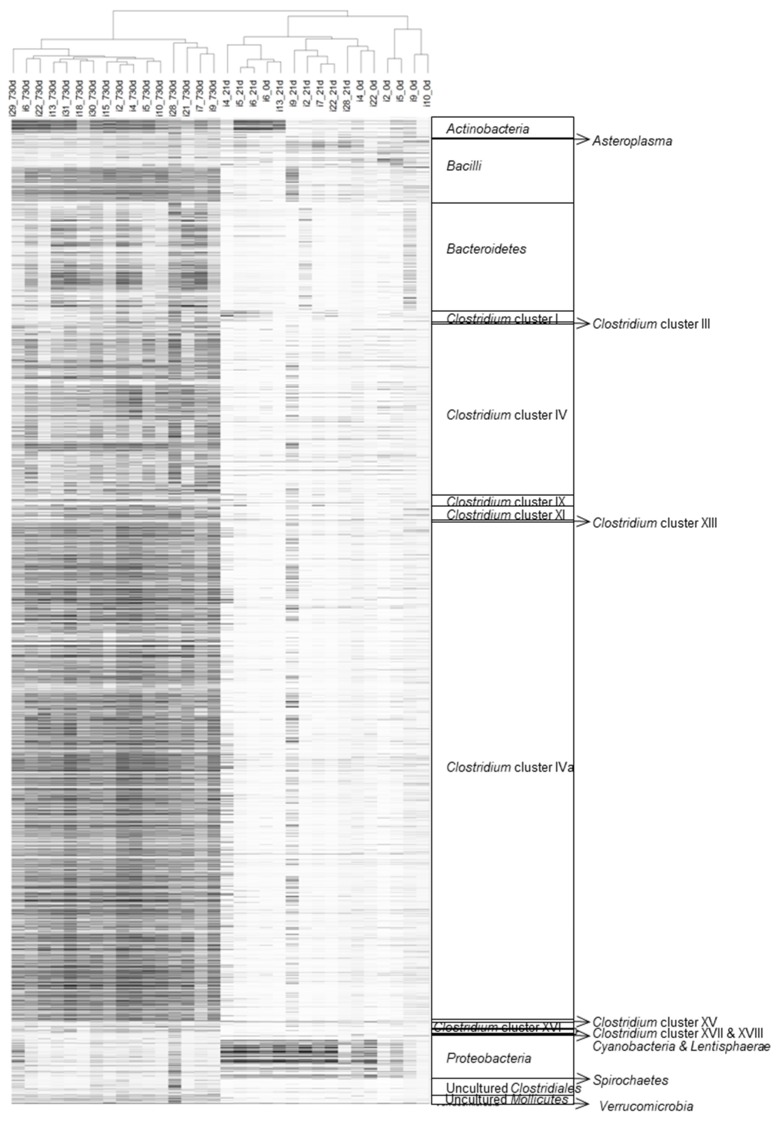
Hierarchical clustering of the intestinal microbiota. Unsupervised clustering was performed for HITChip oligoprofiles, obtained from fecal samples of the preterm infants at meconium (0 days), third week (21 days) and 2-years (720 days). Each line represents a different probe and the darkness of the lines represents the probe abundance in the sample. The highest phylogenetic levels represented are shown on the right side of the figure. Pearson’s correlation and Ward’s clustering methods were used.

The microarray datasets of the 16 fecal samples, collected two years after birth and those previously obtained from meconium and feces, collected in the third week of life [36] were analyzed and hierarchically clustered, based on the signal intensity of the 3699 distinct HITChip oligonucleotide probes (Figure 1, Table S1). The obtained unsupervised microbiota profiles clustered into three different groups, according to the sampling times, except for those of the meconium samples of infants 4, 6 and 22 (Figure 1).

**Figure 2:**
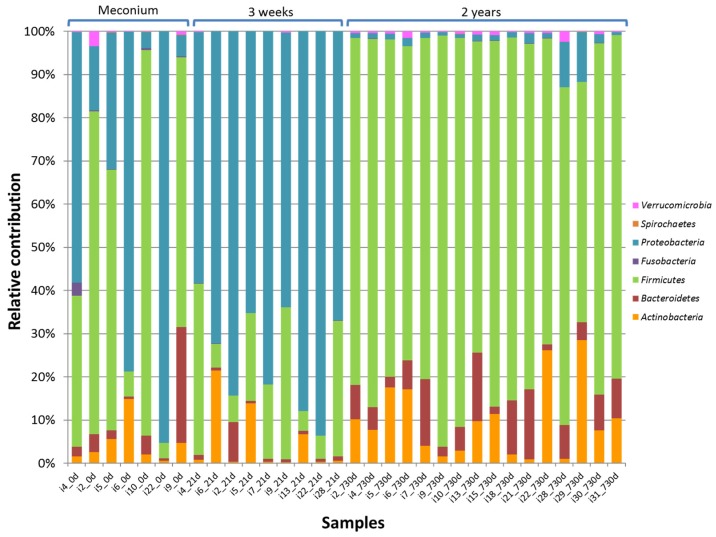
Development of the intestinal microbiota composition at the phylum level. The relative contribution is shown for the phyla detected in meconium, third week and 2-year fecal samples of the preterm infants.

The relative contribution of the major phyla (Table S2) was assessed as the percentage of the phylum taxa among the total microbiome detected in each fecal sample (Figure 2). Globally, approximately two thirds of the DNA phylotypes retrieved from the meconium samples were ascribed to the *Firmicutes* phylum (63.4%). The phylum *Proteobacteria* dominated in the 21-day fecal samples (about 60.0%) but became a minor species in the 2-year samples (2.5%). Interestingly, *Firmicutes* was again the most abundant phylum (78.5%) in the later samples, followed by *Actinobacteria* (9.9%) and *Bacteroidetes* (7.7%).

**Figure 3:**
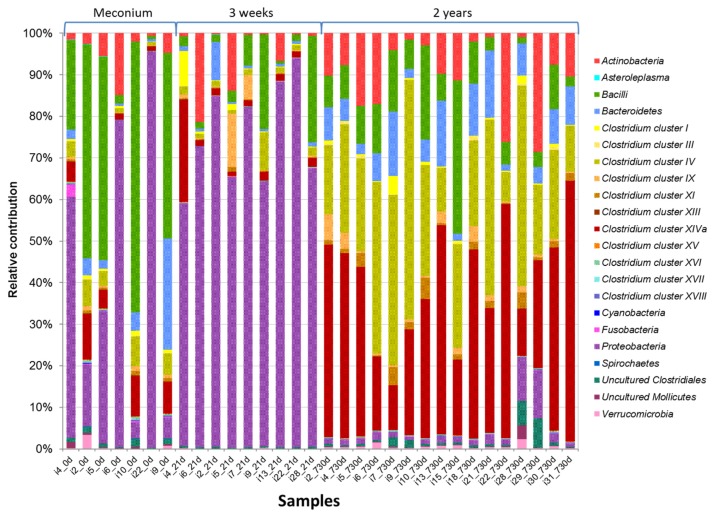
Development of the intestinal microbiota composition at the phylum/order level. The relative contribution is shown for the phylum/order-like phylogroups of the microbiota of meconium, third week and 2-year fecal samples of the preterm, as assessed using the HITChip microarray. Only phylum/order-like phylogroups that contributed at least 0.1% to a given profile are shown.

Within the *Firmicutes*, those belonging to the class *Bacilli* were the most abundant, both in meconium and 21-day feces (18.8%). However, the diversity of *Firmicutes* was notably higher in 2-year fecal samples, where *Clostridium* cluster XIVa (35.2%) and *Clostridium* cluster IV (27.1%) became the predominant classes (Figure 3).

On a lower taxonomic level, the comparison of the 130 hybridization signals, corresponding to the genus-like bacterial groups (Level 2, Table S3), obtained from the 21-day and 730-day fecal samples, showed that 91 phylogenetic groups differed significantly between both types of samples. Among them, 60 phylogenetic groups contributed >0.1% to the microbial profile of each sample (Table 3). The presence of 10 genus-like groups (Level 1) significantly decreased in the 2-year fecal samples, compared to the 21-day ones. It should be highlighted that most of the genera that were significantly reduced (Table 3, positive fold change ↑) are typically associated with hospital environments, including bacteria related to *Enterobacter aerogenes*, *Enterococus* spp. *E. coli*, *Granulicatella* spp., *K. pneumoniae*, and *Proteus*, *Serratia* and *Yersinia* spp. In contrast, there was a very high increase (≥40-fold) from the 21-day to 2-year microbiota, in the abundance of bacteria related to the known carbohydrate degraders, *Bacteroides vulgatus*, *Lactococcus* spp., *Ruminococcus bromii* and *Ruminococcus obeum* as well as the butyrate producers, *Anaerostipes caccae*, *Coprococcus eutactus* and *Eubacterium hallii* (Table 3, negative fold change ↓).

The comparison of genus-like bacterial groups (level 2) obtained from the 730-day fecal samples of preterm infants and 2–4-year-old term infants showed differences in 65 genus-like bacterial groups (Supplementary Materials Table S4) belonging to five different phyla: *Actinobacteria*, *Bacteroidetes*, *Firmicutes*, *Fusobacteria* and *Proteobacteria*. Almost all the *Firmicutes*, the majority butyrate-producing bacteria were more abundant in 2-year-old children born preterm, than those born at term. In contrast, lactic acid bacteria accounted for a higher amount of the hybridization signal in children born at term represented by *Lactobacillus plantarum et rel*. The genus, *Bifidobacterium*, accounted for less than 0.1% of the total hybridization signal in 2-year-old children, born preterm, while in 2–4-year-old children, born at term, levels were high [38].

**Figure 4:**
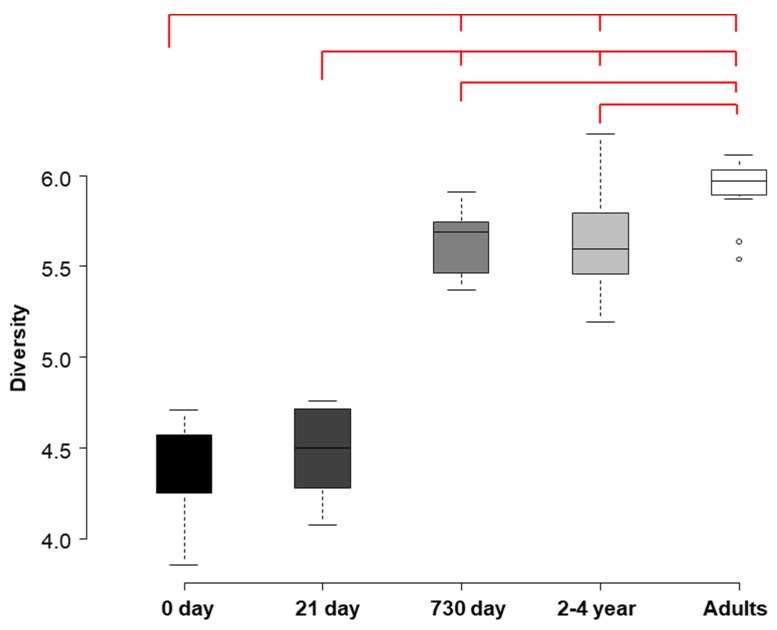
Development of the intestinal microbiota diversity. Shannon–Weaver diversity indices are shown for the intestinal microbiota in the meconium, third week and 2-year fecal samples of preterm infants. Boxes at the right and middle represent the Shannon–Weaver diversity index obtained in meconium, 21-day and 2-year feces. Boxes on the right represent Shannon–Weaver diversity indexes previously obtained with the Human Intestinal Tract Chip (HITChip) in feces, from 2 and 4-year-old healthy infants and from healthy adults. The red bars on the top represent pairs of indices that were found to be statistically different (*p* < 0.001).

The Shannon–Weaver diversity indices, obtained from meconium, 21-day and 2-year fecal samples were compared with data available on the HITChip database for fecal samples from 2–4-year-old healthy infants and, also, from healthy adults (Figure 4). The microbial diversity increased with age, both in preterm and healthy individuals. Diversity indices differed significantly among all the groups, except for (a) the meconium and 21-day samples, and (b) the 2-year samples obtained in this study and the data from 2–4-year-old healthy infants existing in the HITChip database (paired *t*-test; *p* = 0.511 and *p* = 0.957 respectively).

**Figure 5:**
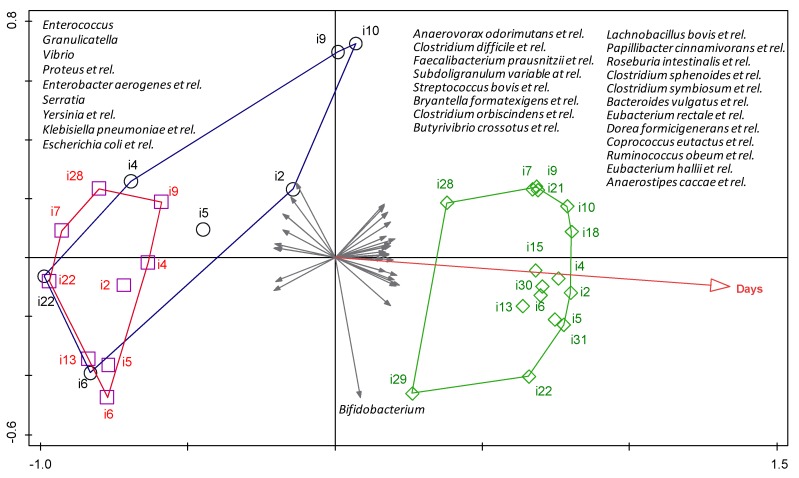
Correlation analysis for the intestinal microbiota. The results of a redundancy analysis are shown for the bacterial composition of meconium (blue circles), third week (red squares) and 2-year (green rhombus) fecal samples of the preterm infants. Arabic numbers indicate the different infants. Gray arrows indicate the bacterial groups associated with the different samples. The plotted first and second ordination axes explained 41.4% of the variability in the data set. Age was the only variable that was significantly related to the sample distribution (*p* = 0.006, Monte Carlo Permutation Testing (MCPT) with forward selection).

The relationships between the observed differences in the bacterial profiles detected in meconium, 21-day and 2-year feces, and several demographic and clinical variables (Table 1 and Table 2), were explored with a multivariate cluster analysis. A redundancy analysis revealed that, among all variables explored, only the age of the infant had a significant effect on the bacterial community composition at the different sampling times (*p* = 0.006, Monte Carlo Permutation Procedure) (Figure 5). More specifically, the observed distribution could explain 41.4% of the total variation in the dataset (Figure 5). Bacterial groups that were positively associated with increased age included bacteria belonging to butyrate-producing species, such as *Butyrivibrio crossotus*, *Eubacterium rectale* and *Eubacterium hallii*. In contrast, the groups that were negatively associated with age included potentially pathogenic Gram-negative bacteria, typically related to hospital environments, such as *Escherichia*, *Klebsiella*, *Serratia* and *Yersinia*. The uniform length of all vectors depicted in Figure 5 indicates that the strength of the correlation was similar for all bacterial species. The length of the hospital stay was also found to be associated (although not significantly) with 21-day fecal samples and with specific bacterial groups (most notably the *Proteobacteria*). None of the other variables significantly influenced the sample separation.

### 3.3. Immunological Analysis

The concentration of a broad range of immune compounds, including cytokines, chemokines, growth factors and immunoglobulins was measured in nine meconium and 9, 15 and 16 fecal samples from the first week, third week and second year of life, respectively. Initially, an exploratory screening was performed, in order to detect outliers; this analysis revealed that the 2-year fecal sample from infant 2 was very different from the rest of the sample set in the PCA analysis of immune compounds. The medical history from this infant reflected a high incidence of acute otitis media during the first two years of life and, in fact, a few days after the collection of this fecal sample, this infant was submitted to an emergency surgery. Therefore, this sample was excluded from the general immunological analysis.

Globally, the values obtained for all these immune factors showed high interindividual variability, in both detection frequencies and concentration. Meconium samples showed a lower presence of immune-related compounds, compared to fecal samples, except for MIP-1β and GM-CSF (Table 4). Most cytokines related to either innate or acquired immunity were detected in less than 50% of the samples, except for IL-1β, in the first and third weeks of life; IL-4 (for which detection frequency increased over time); and IL-17 in the third week and after two years from birth. Among the chemokines, MCP-1 (2-year samples) and MIP-1β (meconium, first and third week fecal samples) were also detected in more than 50% of the samples, while the hematopoietic factors, G-CSF and GM-CSF, were detected in higher numbers (Table 4). All the detection frequencies (except those for IL-6 and IL-13) changed significantly (*p* < 0.018) depending on the sampling time (Table 4). However, only GM-CSF showed a statistically significant difference (*p* = 0.002), with higher levels in meconium and 2-year samples, when the evolution over time of the concentrations of all the immune compounds was considered (Table 4). Some immune compounds (IL-1β, IL-2 and MIP-1β) showed a decreasing tendency, while others (IL-6, IL-12(p70), IFN-γ, TNF-α, MCP-1 and IL-5) tended to increase over time.

The concentrations of IgG1, IgG2, IgG3, IgG4, IgM and IgA in meconium and fecal samples, taken at 7, 21 and 730 days after birth, are shown in Table 5. With the exception of IgA, a high degree of variability and a low frequency of detection were observed for immunoglobulins. Similarly to cytokines, the detection frequencies of all the immunoglobulins changed significantly, depending on the sampling time but no statistically significant differences were found in relation to their concentrations. IgA was the most abundant immunoglobulin in all the tested samples with a significant change (*p* = 0.004) over time. The median IgA concentrations in the first week fecal samples (26.62 mg/g) were approximately 26 times higher than those detected in the meconium ones (1.02 mg/g); the concentration then decreased in the later time samples.

Immune profiles of fecal samples, collected at 21 and 720 days after delivery of preterm infants, were compared with immune profiles of fecal samples collected at 90 and 300 days after delivery of healthy infants born at term. Despite the difference between time of collection of the samples, interesting divergences were observed. Both concentration and frequency of detection of cytokines were higher in fecal samples of preterm infants than in infants born at term. On the other hand, the frequency and the concentration of immunoglobulins were higher in fecal samples of infants born at term than preterm, except for IgA.

**Figure 6:**
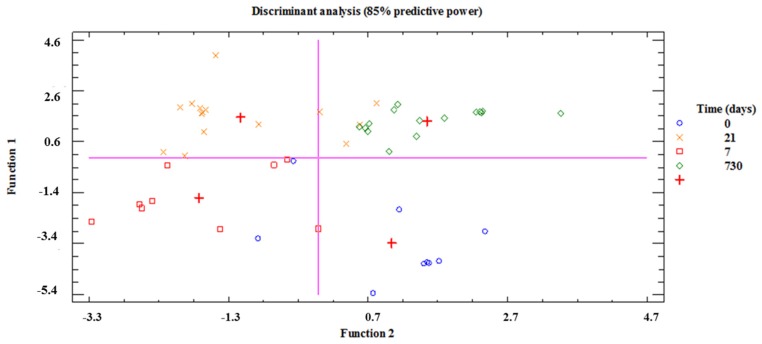
Multiple discriminant analysis (MDA) of the immune data. MDA was applied to the immunological data of meconium (blue circles), first week (red squares), third week (orange cross), and 2-year (green rhombus) feces, taking sampling time as the discriminant factor. The red cross represents the mathematical centroid for each sampling time group (meconium, first week, third week, and 2-year fecal samples). The first and second functions, which were plotted as the *x* and *y* axes, had a predictive power of 85% and internal axes (pink lines) match the zero values for both functions.

When a multiple discriminant analysis (MDA) was applied to all the variables, taking sampling time as the discriminant factor, two derivative functions with an eigenvalue >1 were obtained. The predictive power of the MDA was 85.42% and the expression of the first derivative function (Function 1) had a canonical correlation factor of 0.9018 that was statistically significant (*p* = 0.002). The standardized coefficients for this function were the following: IL-1b (−0.96), IL-2 (−3.38), IL-4 (1.22), IL-5 (−0.56), IL-6 (−0.48), IL-7 (−1.72), IL-8 (−2.81), IL-10 (−4.56), IL-12(p70) (4.50), IL-13 (−1.10), IL-17 (−5.61), G-CSF (4.38), GM-CSF (−0.67), IFN-γ (4.49), MCP-1 (0.81), MIP-1β (8.88), TNF-α (1,60), GRO-α (−4.78), IgG_1_ (2.13), IgG_2_ (−6.37), IgG_3_ (−2.71), IgG_4_ (−0.50), IgM (0.36), IgA (0.30). In fact, the MDA representation allowed a differential classification of fecal samples, according to infant age, when considering their profiles of immune compounds (Figure 6). Furthermore, the centroid of each group of samples (meconium, first week, third week and 2-year feces) was located in a different quadrant of the coordinates delimitated by the zero values of the axes (Figure 6).

## 4. Discussion

In this study, the bacterial compositions of fecal samples obtained from 2-year-old infants that were born preterm were assessed and compared to those from meconium and third week of life fecal samples obtained from the same infants [36,40,41]. In addition, a wide range of cytokines, chemokines, growth factors and immunoglobulins were determined in all the meconium and fecal samples, in order to describe their immunological profiles, their changes over time and their potential relationships with bacterial colonization.

The results obtained from meconium and third week fecal samples showed a low diversity of bacterial species and high interindividual variability, while the opposite was observed in those taken from the same infants at the age of 2. Globally, the bacterial communities evolved towards an adult-like microbiota, which is the normal evolution of the microbiome of healthy term infants as they age [9,42]. Fecal samples taken 2 years after birth showed a distinctive bacterial composition when compared to that obtained from the same infants when they were 3-weeks-old. Those genera, related with a hospital environment—such as *E. coli*, *Klebsiella* or *Serratia*—and present in the third week of life seemed to be replaced, two years later, by genera belonging to *Clostridium* clusters, IV and XIVa. The predominance of such genera is a feature of the healthy adult gut microbiome, as a part of a complex microbiota, which is characterized by slow turnover, preference for low redox potential and high production of short chain fatty acids [43].

In this study, bacterial diversity increased with age, in agreement with previous works that have reported that the number of operational taxonomic units (OTUs) detected in fecal samples increases with age in different human populations [9]. The Shannon diversity index of the microbiota, present in 2-year-old infant stools was higher than that observed in the 21-day ones, similar to that calculated from the 2–4-year-old healthy infants from the HITChip database, and lower to that of healthy adults, as deduced using the same database. Remarkable changes occur in the gut colonization pattern throughout the first two or three years of life, but then, the microbiota stabilizes and starts to resemble that of adulthood [8,44]. The results of this study indicate that the diversity of the gut microbiota of 2-year-old infants, who were born preterm, has not yet reached the attributes of the adult microbiome.

Colonization of the infant gut by *Lactobacilli* and *Bifidobacteria* is often delayed or even absent in the case of antibiotic-treated infants [12,15,45], including preterm neonates [36]. The results obtained in this study indicate that this may be a long-lasting effect of prematurity, since, after two years from birth, the relative abundance of *Lactobacilli* was still low, compared to age-matched term infants (Supplementary Materials Table S4).

Previous studies, focused on the detection and quantification of cytokines, chemokines and immunoglobulins in blood samples, from term and preterm infants, have shown that there are differences in their immune profiles, depending on their gestational age [27,29,31]. However, as far as we know, this is the first study where a wide range of immune compounds has been assessed in the meconium and feces of preterm infants and followed up when they were 2 years old. Interestingly, each type of sample (meconium, 7-day, 21-day and 2-year feces) showed a different immune pattern and, in fact, the MDA analysis, performed with all the immune variables, exhibited a high predictive power, highlighting the differences in the immune profiles among the different sampling times.

In this study, the median IgA concentration increased notably from meconium to first week feces but in the third week of life, there was a progressive reduction, although not significant, reaching levels similar to those of healthy infants [46,47]. This probably results from the massive arrival of bacteria and other antigens to the gut after birth, since microbial gut colonization triggers the production of IgA by the gut-associated lymphoid tissue (GALT) [48]. This high IgA concentration in the first weeks of life became lower in the 2-year samples. In addition, lactating mammary glands are part of the secretory immune system, and IgA antibodies in breast milk reflect the antigenic stimulation of mucosal-associated lymphoid tissue [49]. Breast-milk antibodies are, thus, highly targeted against infectious agents and other exogenous antigens in the mother’s environment, which are those likely to be encountered by the infant [32]. Therefore, breastfeeding represents an ingenious immunologic mother–infant integration [49,50,51,52]. This fact highlights the importance of the availability of own mother’s or donor’s milk to feed preterm neonates, a population particularly sensitive to infectious and inflammatory diseases. It should be noted that an abnormal gut microbial colonization predisposes the neonatal intestine to inflammation and to a cascade of pro-inflammatory and anti-inflammatory cytokine responses [53]. The ability of IgA to penetrate the gut mucosal surface, in conjunction with antigens and, as a consequence, to induce effector immune responses, plays a key role in the maintenance of intestinal microbiota and immune homeostasis [54].

The comparison of the immune profiles of fecal samples of preterm infants, collected at 720 days after delivery, with samples from infants born at term, collected at day 300, suggested a higher activity of B lymphocytes in the latter. This fact may be associated with a higher secretory maturity, whereas 2-year-old children born preterm have a higher activity of mediators of the immune system, which may be associated with a high activity of T lymphocytes.

Work is in progress to characterize the wide collection of bacterial isolates obtained from the biological samples analyzed in this study and, therefore, to elucidate at the strain or clone level if initial colonizers may persist later in life.

## 5. Conclusions

Hospital-associated fecal bacteria, dominant during NICU stay are replaced, two years later, by adult-like genera. In contrast to infants born at term, preterm infants have a low abundance of *lactobacilli* and *bifidobacteria* at two years of age. The immune profiles of the meconium and fecal samples differed, depending on the sampling time, showing different immune maturation statuses of the gut.

## Figures and Tables

**Table 1:** Demographic data for the infant cohort.

Infant	Gestational Age (week)	Delivery Mode	Gender	Birth Weight (g)
2	30	Caesarean section	Male	1550
4	27	Caesarean section	Female	1080
5	30	Caesarean section	Male	2030
6	30	Vaginal	Male	1760
7	24	Caesarean section	Female	600
9	27	Vaginal	Male	1540
10	26	Caesarean section	Female	790
13	32	Vaginal	Female	1310
15	30	Vaginal	Female	1350
18	24	Vaginal	Male	740
21	28	Caesarean section	Male	1100
22	31	Vaginal	Female	1430
28	27	Vaginal	Female	1040
29	29	Caesarean section	Male	680
30	30	Vaginal	Female	1370
31	28	Caesarean section	Female	1150

**Table 2:** Clinical characteristics of the preterm infants recruited in this study.

Infant	Hospital Stay (days)	Antibiotic Therapy (days)	Mechanical Ventilation (days)	Parenteral Nutrition (days)	Nasogastric Feeding Tube (days)	Meconium Expulsion (h)
2	42	3	0	5	38	59
4	60	4	0.5	3	48	24
5	27	3	2	0	26	11
6	27	0	0	0	26	7
7	113	5	9	8	107	116
9	68	4	2	5	58	96
10	84	7	0.5	6	70	0
13	28	3	0	0	21	12
15	44	3	1	7	38	11
18	116	27	35	13	112	144
21	73	7	10	14	62	48
22	37	3	0	4	35	3
28	68	7	0	7	62	12
29	70	15	8	9	60	144
30	41	4	0	3	40	
31	52	3	0	6	47	9
Mean (95% CI)	58.64 (41.35; 75.93)			4.85 (2.66; 7.05)	52.40 (36.03; 68.82)	46.4 (17.22;75.58)
Median (IQR)		3.5 (3.0–7.0)	0.25 (0.00–1.75)			

CI: Confidence interval; IQR: Inter-quartile range.

**Table 3:** Relative counts of genus-like bacterial groups. The genus-like phylogenetic groups (Level 2; [39]), detected in fecal samples, collected at 21 days and 2 years after birth, from preterm infants, are shown ^a^.

Phylum/Order	Genus-Like Phylogenetic Group ^¥^	21 Days Mean (95% CI)	730 Days Mean (95% CI)	*p*-Value *	Fold Change #
*Actinobacteria*	*Collinsella*	0.04 (0.03; 0.04)	0.25 (0.18; 0.32)	0.009	↑6.77
*Bacteroidetes*	*Allistipes et rel.*	0.09 (0.08; 0.11)	0.38 (0.17; 0.59)	0.009	↑4.06
	*Bacteroides ovatus et rel.*	0.06 (0.05; 0.07)	0.38 (0.05; 0.71)	0.009	↑6.69
	*Bacteroides plebeius et rel.*	0.04 (0.03; 0.04)	0.12 (0.08; 0.16)	0.009	↑3.23
	*Bacteroides splachnicus et rel.*	0.09(0.08; 0.11)	0.26 (0.11; 0.41)	0.016	↑2.73
	*Bacteroides stercoris et rel.*	0.04 (0.03; 0.04)	0.11 (0.05; 0.16)	0.009	↑2.97
	*Bacteroides vulgatus et rel.*	0.08 (0.05; 0.09)	3.12 (1.54; 4.70)	0.009	↑39.50
	*Parabacteroides distasonis et rel.*	0.06 (0.05; 0.07)	0.32 (0.16; 0.48)	0.009	↑5.20
	*Prevotella melaninogenica et rel.*	0.11 (0.09; 0.13)	0.88 (−0.16; 1.92)	0.033	↑8.20
	*Prevotella oralis et rel.*	0.04 (0.03; 0.04)	0.24 (−0.02; 0.50)	0.016	↑6.55
	*Prevotella tannerae et rel.*	0.02 (0.02; 0.03)	0.10 (0.07; 0.13)	0.009	↑4.20
	*Tannerella et rel.*	0.05 (0.04; 0.06)	0.13 (0.11; 0.16)	0.009	↑2.63
*Bacilli*	*Enterococcus*	3.49 (−1.05; 8.03)	0.10 (0.06; 0.13)	0.009	↓36.18
	*Granulicatella*	1.00 (0.19; 1.80)	0.02 (0.01; 0.02)	0.009	↓61.31
	*Lactococcus*	0.02 (0.01; 0.02)	0.73 (−0.02; 1.48)	0.056	↑41.05
	*Staphylococcus*	0.19 (−0.05; 0.43)	0.02 (nd)	0.033	↓10.86
	*Streptococcus intermedius et rel.*	0.04 (0.02; 0.05)	0.15 (0.11; 0.20)	0.033	↑3.96
*Clostridium* cluster III	*Clostridium stercorarium et rel.*	0.03 (0.03; 0.04)	0.28 (0.15; 0.41)	0.009	↑9.26
*Clostridium* cluster IV	*Clostridium cellulosi et rel.*	0.13 (0.11; 16)	3.49 (1.20; 5.79)	0.009	↑26.48
	*Clostridium leptum et rel.*	0.18 (0.09; 0.28)	2.16 (0.74; 3.57)	0.009	↑11.81
	*Clostridium orbiscindens et rel.*	1.03 (−0.68; 2.73)	5.59 (3.90; 7.29)	0.042	↑5.44
	*Faecalibacterium prausnitzii et rel.*	0.17 (0.14; 0.20)	5.02 (1.81; 8.22)	0.009	↑29.35
	*Oscillospira guillermondii et rel.*	0.15 (0.13; 0.18)	3.10 (−0.13; 6.32)	0.009	↑20.04
	*Papillibacter cinnamivorans et rel.*	0.06 (0.05; 0.07)	0.50 (0.34; 0.66)	0.009	↑8.56
	*Ruminococcus bromii et rel.*	0.02 (0.02; 0.03)	1.31 (0.20; 2.43)	0.009	↑52.65
	*Ruminococcus callidus et rel.*	0.08 (0.07; 0.09)	0.86 (−0.06; 1.78)	0.009	↑10.57
	*Sporobacter termitidis et rel.*	0.16 (0.14; 0.19)	1.89 (0.96; 2.81)	0.009	↑11.56
	*Subdoligranulum variable at rel.*	0.11 (0.09; 0.13)	2.98 (1.48; 4.48)	0.009	↑27.51
*Clostridium* cluster IX	*Dialister*	0.05 (0.02; 0.07)	0.75 (−0.03; 1.54)	0.009	↑16.10
*Clostridium* cluster XI	*Anaerovorax odorimutans et rel.*	0.05 (0.04; 0.06)	0.41 (0.27; 0.55)	0.009	↑7.79
*Clostridium* cluster XIVa	*Anaerostipes caccae et rel.*	0.09 (0.05; 0.12)	3.94 (2.10; 5.77)	0.009	↑45.14
	*Bryantella formatexigens et rel.*	0.14 (0.08; 0.21)	0.69 (0.50; 0.89)	0.009	↑4.82
	*Butyrivibrio crossotus et rel.*	0.16 (0.09; 0.23)	1.41 (1.14; 1.68)	0.009	↑8.89
	*Clostridium colinum et rel.*	0.05 (0.04; 0.06)	0.14 (0.06; 0.23)	0.009	↑2.77
	*Clostridium sphenoides et rel.*	0.12 (0.09; 0.14)	0.85 (0.56; 1.14)	0.009	↑7.38
	*Clostridium symbiosum et rel.*	0.22 (0.13; 0.32)	1.81 (1.38; 2.24)	0.009	↑8.09
	*Coprococcus eutactus et rel.*	0.08 (0.06; 0.09)	4.96 (2.87; 7.04)	0.009	↑64.93
	*Dorea formicigenerans et rel.*	0.16 (0.08; 0.23)	2.63 (1.86; 3.40)	0.009	↑16.72
	*Eubacterium hallii et rel.*	0.04 (0.02; 0.06)	2.23 (1.40; 3.05)	0.009	↑52.41
	*Eubacterium rectale et rel.*	0.06 (0.04; 0.08)	0.42 (0.26; 0.58)	0.009	↑7.09
	*Eubacterium ventriosum et rel.*	0.05 (0.02; 0.07)	0.28 (0.13; 0.42)	0.009	↑6.00
	*Lachnobacillus bovis et rel.*	0.07 (0.05; 0.07)	0.31 (0.26; 0.36)	0.009	↑4.43
	*Lachnospira pectinoschiza et rel.*	0.10 (0.07; 0.13)	0.40 (0.28; 0.53)	0.009	↑4.08
	*Roseburia intestinalis et rel.*	0.03 (0.01; 0.05)	0.24 (0.15; 0.34)	0.009	↑7.86
	*Ruminococcus lactaris et rel.*	0.03 (0.02; 0.03)	0.42 (−0.18; 1.02)	0.009	↑16.30
	*Ruminococcus obeum et rel.*	0.26 (0.21; 0.31)	11.42 (8.18; 14.66)	0.009	↑43.96
Uncultured *Clostridiales*	Uncultured *Clostridiales I*	0.17 (0.14; 0.20)	1.11 (0.12; 2.10)	0.009	↑6.48
	Uncultured *Clostridiales II*	0.18 (0.16; 0.21)	0.36 (0.21; 0.51)	0.009	↑1.97
Uncultured *Mollicutes*	Uncultured *Mollicutes*	0.08 (0.07; 0.10)	0.32 (−0.07; 0.70)	0.022	↑3.94
*Proteobacteria*	*Burkholderia*	0.01 (nd)	0.10 (−0.04; 0.24)	0.016	↑10.98
	*Enterobacter aerogenes et rel.*	14.82 (8.07; 21.57)	0.21 (0.10; 0.32)	0.009	↓69.90
	*Escherichia coli et rel.*	37.06 (24.37; 49.76)	0.82 (−0.25; 1.88)	0.009	↓45.41
	*Klebisiella pneumoniae et rel.*	15.75 (10.18; 21.32)	0.11 (0.05; 0.18)	0.009	↓138.34
	*Oxalobacter formigenes et rel.*	0.02 (0.02; 0.03)	0.17 (0.02; 0.32)	0.009	↑7.35
	*Proteus et rel.*	0.29 (0.17; 0.42)	0.09 (0.05; 0.12)	0.009	↓3.46
	*Serratia*	5.18 (1.88; 8.49)	0.15 (−0.04; 0.33)	0.009	↓35.00
	*Sutterella wadsworthia et rel.*	0.08 (0.07; 0.10)	0.23 (0.07; 0.39)	0.009	↑2.79
	*Vibrio*	0.11 (0.08; 0.15)	0.04 (0.03; 0.04)	0.009	↓2.94
	*Yersinia et rel.*	1.16 (0.63; 1.68)	0.04 (0.03; 0.04)	0.009	↓31.05
*Verrucomicrobia*	*Akkermansia*	0.06 (0.00; 0.13)	0.62 (0.34; 0.91)	0.016	↑9.64

^a^ Relative counts (log-transformed hybridization signals) are expressed as the mean and 95% confidence interval. nd, no data. ^¥^ The genus-like phylogenetic groups shown, contributed at least 0.1% to the microbial profile of a given sample. * *t*-tests were used to evaluate differences in the hybridization signal intensities of genus-like bacterial groups across time. # Fold changes were calculated as log-transformed hybridization signals at 2 years over those at 3 weeks.

**Table 4:** Immunological analysis of intestinal samples. The presence and concentrations are shown for cytokines and other immune compounds in the meconium and fecal samples collected in this study.

	Meconium (*n* = 9)		Feces (7 days) (*n* = 9)		Feces (21 days) (*n* = 15)		Feces (2 years) (*n* = 15)		*p*-Value ^♯^	*p*-Value ^ǂ^
	*n* (%) ^1^	Median (IQR)	*n* (%)	Median (IQR)	*n* (%)	Median (IQR)	*n* (%)	Median (IQR)		
Innate immunity										
IL-1β	2 (22.22)	1.24 (0.68–1.80)	7 (77.78)	0.13 (0.05–0.30)	13 (86.67)	0.05 (0.03–0.38)	0	-	0.000	0.520
IL-6	1 (11.11)	0.03	2 (22.22)	0.02 (0.02–0.03)	3 (20.00)	0.05 (0.05–0.71)	4 (26.67)	0.3 (0.15–0.47)	0.094	0.255
IL-12p70	1 (11.11)	0.06	2 (22.22)	0.11 (0.08–0.15)	4 (26.67)	0.07 (0.05–0.12)	4 (26.67)	0.84 (0.28–1.38)	0.018	0.601
IFN-γ *	0	-	0	-	1 (6.67)	3.27	3 (20.00)	9.27 (−7.44; 25.98)	0.000	0.521
TNF-α	1 (11.11)	0.14	2 (22.22)	0.15 (0.13–0.17)	5 (33.33)	0.18 (0.10–0.25)	3 (20.00)	0.27 (0.17–0.43)	0.002	0.934
Acquired immunity										
IL-2	1 (11.11)	0.92	1 (11.11)	0.09	2 (13.33)	0.04 (0.03–0.05)	4 (26.67)	0.04 (0.02–0.15)	0.003	0.373
IL-4	4 (44.44)	0.01 (0.00–0.01)	6 (66.67)	0.01 (0.00–0.01)	11 (73.33)	0.01 (0.00–0.01)	15 (100.00)	0.01 (0.00–0.01)	0.000	0.292
IL-10	0	-	1 (11.11)	0.03	1 (6.67)	0.04	4 (26.67)	0.03 (0.02–0.23)	0.000	0.675
IL-13 *	1 (11.11)	0.03	1 (11.11)	0.05	2 (13.33)	0.08 (−0.10; 0.26)	1 (6.67)	0.05	0.568	0.571
IL-17	4 (44.44)	3.09 (2.32–3.31)	2 (22.22)	0.08 (0.05–0.12)	9 (60.00)	0.07 (0.03–0.19)	12 (80.00)	0.08 (0.04–0.06)	0.000	0.122
Chemokines										
IL-8 *	4 (44.44)	0.07 (−0.08; 0.21)	1 (11.11)	0.05	3 (20.00)	0.09 (−0.02; 0.20)	0	-	0.000	0.865
MCP-1	3 (33.33)	0.03 (0.02–0.03)	1 (11.11)	0.03	4 (26.67)	0.05 (0.03–0.06)	9 (60.00)	0.19 (0.06–0.88)	0.000	0.080
MIP-1β	6 (66.67)	0.62 (0.05–1.78)	6 (66.67)	0.05 (0.04–0.16)	8 (53.33)	0.07 (0.02–0.10)	2 (13.33)	0.05 (0.04–0.07)	0.000	0.639
GRO-α	3 (33.33)	0.18 (0.16–8.28)	0 (0.00)	-	6 (40.00)	0.16 (0.11–0.19)	3 (20.00)	0.14 (0.13–0.22)	0.000	0.537
Hematopoyetic factors										
IL-5 *	0	-	0	-	1 (6.67)	0.03	5 (33.33)	0.25(0.03; 0.47)	0.000	0.322
IL-7	1 (11.11)	0.03	1 (11.11)	0.04	3 (20.00)	0.03 (0.02–0.05)	0	-	0.000	0.766
G-CSF	4 (44.44)	1.64 (0.34–4.29)	6 (66.67)	0.06 (0.04–0.17)	12 (80.00)	0.28 (0.15–0.55)	12 (80.00)	0.14 (0.11–0.46)	0.000	0.220
GM-CSF	9 (100.00)	0.59 ^a^ (0.28–55.03)	7 (77.78)	0.23 ^ab^ (0.12–0.40)	15 (100.00)	0.23 ^ab^ (0.18–0.26)	15 (100.00)	0.67 ^ac^ (0.62–0.87)	0.000	0.002

Concentrations (ng/g feces) are expressed as median and interquartile range (IQR). ^1^ *n* (%): number of samples in which the parameter was detected (relative frequency of detection). ^♯^ Chi-squared tests were used to evaluate differences in expression frequencies of the analyzed parameters. **^ǂ^** One-way ANOVA or Kruskal–Wallis tests were used to evaluate differences in concentration across time. Different superscript letters show which medians were different within groups. * These data sets were normally distributed and concentrations are expressed as mean and 95% CI.

**Table 5:** Detection and concentrations of immunoglobulins (Ig) in the meconium and fecal samples collected in this study.

	Meconium (*n* = 9)		Feces (7 days) (*n* = 9)		Feces (21 days) (*n* = 15)		Feces (2 years) (*n* = 15)		*p*-Value ^♯^	*p*-Value ^ǂ^
	*n* (%) ^1^	Median (IQR)	*n* (%)	Median (IQR)	*n* (%)	Median (IQR)	*n* (%)	Median (IQR)		
IgG1	4 (44.44)	0.03 (0.02–0.06)	2 (22.22)	0.34 (0.18–0.50)	3 (20.00)	0.32 (0.17–0.76)	1 (6.67)	0.06	0.000	0.621
IgG2	6 (66.67)	1.54 (0.84–3.39)	7 (77.78)	0.99 (0.84–1.19)	3 (20.00)	0.35 (0.27–0.62)	2 (13.33)	0.34 (0.24–0.44)	0.000	0.062
IgG3 *	0	-	1 (11.11)	0.01	0	-	2 (13.33)	0.01 (−0.01; 0.03)	0.000	0.326
IgG4 *	2 (22.22)	0.00 (−0.01; 0.01)	3 (33.33)	0.01 (−0.00; 0.02)	1 (6.67)	0.00	1 (6.67)	0.01	0.000	0.510
IgM	1 (2.08)	0.55	6 (12.50)	2.27 (0.53–5.14)	11 (22.92)	0.54 (0.33–0.88)	1 (6.67)	0.08	0.000	0.368
IgA	5 (55.56)	1.02 ^a^ (0.48–67.45)	8 (88.89)	26.62 ^ab^ (10.42–58.88)	15 (100.00)	6.24 ^ab^ (3.69–26.13)	13 (86.67)	1.13 ^ac^ (0.81–3.18)	0.000	0.004

Concentrations (mg/g feces) arere expressed as median and interquartile range (IQR). ^1^ *n* (%): number of samples in which the parameter was detected (relative frequency of detection). ^♯^ Chi-squared tests were used to evaluate differences in expression frequencies of the analyzed parameters. **^ǂ^** One-way ANOVA or Kruskal–Wallis tests were used to evaluate differences in concentration across time. Different superscript letters show which medians were different within groups. * These data sets were normally distributed and concentrations are expressed as mean and 95% CI.

## Supplementary Materials

The following are available online at http://www.mdpi.com/2072-6643/9/12/1293/s1, Table S1: L1 HITChip composition, Table S2: L2 HITChip composition, Table S3: Oligoprofile HITChip composition, Table S4: Relative counts of significant different genus-like bacterial groups of preterm and at term 2-year-old children (*p* < 0.05).
